# The effect of paste composition, aggregate mineralogy and temperature on the pore solution composition and the extent of ASR expansion

**DOI:** 10.1617/s11527-022-02015-6

**Published:** 2022-08-26

**Authors:** Mahsa Bagheri, Barbara Lothenbach, Karen Scrivener

**Affiliations:** 1 Laboratory of Construction Materials, EPFL, 1015 Lausanne, Switzerland; 2 Concrete and Asphalt Laboratory, Empa, Swiss Federal Laboratories for Materials Science and Technology, 8600 Dübendorf, Switzerland

**Keywords:** Alkali silica reaction, Pore solution composition, Aggregate effect, Paste effect, Temperature effect, ASR expansion

## Abstract

The reaction kinetics of the alkali silica reaction depends on the composition of the pore solution. The evolution of the pore solution composition in different cement pastes and concretes was studied. Pastes containing silica fume or metakaolin had the lowest amount of alkalis in the pore solution. In addition, metakaolin increased the aluminium concentrations. The lowest expansion was measured for the concretes made of blended cement pastes with low alkali and hydroxide content in their pore solution, for the duration of the present study, no additional aluminium effect was observed due to the already low pH. Addition of 400 mM of Li slowed down expansion rate of concrete prisms at 40 and 60 °C, however, similar expansion was observed for samples with and without Li at 60 °C after 1 year. Temperature, alkali concentration and pH of pore solution all have a major effect on ASR expansion.

## Introduction

The alkali silica reaction (ASR) [1] is a major durability issue affecting concrete. The alkaline pore solution dissolves silica-containing minerals within reactive aggregates [2]. The reaction of dissolved silica from aggregates with alkali hydroxides and calcium in the pore solution produces ASR products, which causes cracking and expansion in concrete. The pore solutions of blended cements contain various elements including alkalis (mainly sodium and potassium), hydroxide, calcium, sulfate, silicon, and aluminium. The hydroxide concentration depends mainly on the concentrations of sodium and potassium [3]. The type of cement and supplementary cementitious material (SCM) and their amount affect the composition of the pore solution [4]. SCMs have been widely shown to reduce ASR expansion [5–8]; Al-rich SCMs, for instance, fly ash or metakaolin, have been reported to be more effective in preventing ASR [7–10] than ones containing silica alone (e.g. silica fume). In addition, the aggregate mineralogy has an important influence on the amount of SCM needed to control ASR expansion [11]. Several researchers reported that the dissolution of aggregates releases alkali into the pore solution [11–17], which was also used to develop a test to evaluate the alkali contribution originating from the aggregates [18]. However, separate analysis of cement and aggregate can lead to unecessary elimination of certain aggregate types and/or to unrealistic requirements for the alkali content of the cement [19]. Measuring the composition of the pore solution extracted from the concrete will reveal directly to what extent alkalis are released from aggregates and remain in the pore solution to drive ASR expansion. In addition, systematic pore solution analysis can provide fundamental insights into the main drivers of ASR reaction and a reliable method to assess aggregate reactivity before using them in the new concrete structures.

Lithium was reported to control ASR by McCoy and Caldwell already in 1951 [20]. Different mechanisms of suppression of ASR expansion by Li such as the formation of a non-expansive solid, or the formation of a physical barrier have been suggested [21–23], although the findings reported in literature are contradictory and circumstantial. Lithium has been found to lower the CaO/SiO_2_ and (Na + K)/Si in the ASR product, which has been assumed to make it non-expansive [21, 24]. The smaller ionic radius of Li^+^ and its higher charge density have been proposed as the main reason for the preferential incorporation of Li in ASR products in comparison with K^+^ and Na^+^ [21, 24]. The amount of lithium needed to control ASR depends on different factors such as the amount and availability of other alkalis (Na + K) and the aggregate mineralogy [20, 22, 24, 25]. The presence of calcium and lithium seems to slow down the dissolution kinetics of the aggregates [22, 26]. Some studies also reported that lithium alone decreased silica dissolution [2, 27], which may be confused by the formation of Li_2_SiO_3_ during the measurements [26, 28]. Recent results showed that lithium in the absence of Ca^2+^increases the dissolution of silica and feldspars at high pH values [28].

The aim of the present study was to investigate the relationship between pore solution composition and the extent of ASR expansion. The poresolution compositions of different pastes and their evolution over time were measured at different temperatures to assess how different SCMs types and substitution levels change pore solution composition over 18 months. A study of the pore solution composition from concrete samples with different aggregates was carried out to determine how the aggregate mineralogy affects pore solution composition. In additional experiments, concrete samples were exposed in their own simplified pore solutions (based on the measured pore solution compositions) to avoid any leaching during the ASR expansion test, following the method reported in [8, 29].

## Materials and methods

### Materials

Paste and concrete samples were made based on CEM I/42.5 (Na_2_O_eq_ of 0.79%) Portland cement (PC). Different blended cement pastes (Table 1) were prepared using supplementary cementitious materials, limestone (LS) (Omya, Durcal 5), coarse fly ash (FA-c, 3080 cm^2^/g), and fine fly ash (FA-f, 5070 cm^2^/g), metakaolin (MK) (Burgess), micro silica (SF) (Grade 983-U, Elkem Materials). Table 2 shows the oxide compositions of the materials used based on X-ray fluorescence (XRF) analysis. Gypsum (Gyp, Merck) was added to correct the sulfate balance using isothermal calorimetry experiments for the mixtures containing metakaolin (0.5% of gypsum). For the system containing Li, a certain amount of LiCl (99.9%, Apollo Scientific) was added to the water to reach 400 mmol/L of Li, and for the boosted system, the required amount of NaOH was added to the water to reach 1.09% Na_2_O_eq_ before mixing cement and water. A small amount (0.9–2 l/m^3^) of acrylic superplasticizer (Dynamon SR 914–CH) was added to the mixtures containing more than 10 weight percent of metakaolin. Paste samples were cast with a water to binder ratio of 0.46 or 0.6, and after mixing at 900 rpm/min for 3–4 min. De-ionized water was used to cast paste samples.

**Table 1:** Mix design of the blended cement pastes and the aggregates used to prepare concrete samples

Sample	Mass percentage								Concentration (mmol/L)	Aggregate				
	PC^(a)^	MK^(b)^	FA-f^(c)^	FA-c^(d)^	LS^(e)^	SF^(f)^	Gyp^(g)^	Na_2_O_eq_^(h)^	LiCl^(i)^	U^(j)^	B^(k)^	P^(l)^	Ben^(m)^	Cal^(n)^
PC	100									*	*	*	*	*
MK5	94.5	5					0.5							
MK10	89.5	10					0.5							
MK15	84.6	14.9					0.5							
MK20	79.6	19.9					0.5							
FA-f	70		30											
FA-c	70			30										
LS	70				30									
LC^3^	69.7	14.9			14.9		0.5					*	*	*
SF	85					15								
SFLS	70				15	15								
LiPC	100								400					
LiLC^3^	69.7	14.9			14.9		0.5		400					
LiSFLS	70				15	15			400					
AlkPC	100							1.09						
AlkMK10	89.5	10					0.5	1.09						
AlkMK15	84.6	14.9					0.5	1.09						
AlkMK20	79.6	19.9					0.5	1.09						

^a^Portland cement CEM I/42.5 (Na_2_O_eq_ of 0.79%)

^b^Metakaolin (Burgess)

^c^Fine fly ash (FA-f, 5070 cm^2^/g)

^d^Coarse fly ash (FA-c, 3080 cm^2^/g)

^e^Limestone (Omya, Durcal 5)

^f^Micro silica (Grade 983-U, Elkem Materials)

^g^Gypsum (Merck)

^h^Boosted to 1.09% Na_2_O_eq_ by adding NaOH

^i^LiCl (99.9%, Apollo Scientific)

^j^Swiss alpine aggregates from Switzerland [30]

^k^Bend aggregate from the US

^l^Calcite-based non-ASR reactive aggregate

**Table 2:** XRF oxide composition of the materials

Oxide wt%	PC^(b)^	FA-c^(c)^	LS^(d)^	MK^(e)^	SF^(f)^
SiO_2_	20.1	59.7	0.1	52.0	99
Al_2_O_3_	4.6	23.3	0.001	43.8	–
TiO_2_	0.4	1.1	0.01	1.5	
MnO	0.05	0.03	0.003	0.01	
Fe_2_O_3_	3.3	6.5	0.04	0.3	
CaO	63.0	1.6	55.0	0.03	
MgO	1.8	1.0	0.1	0.01	
K_2_O	1.0	2.0	0.01	0.1	
Na_2_O	0.2	0.5	0.1	0.3	
SO_3_	3.3	0.2	0.03	0.1	
P_2_O_5_	0.2	0.2	0.004	0.2	
Cr_2_O_3_	0.01	–	0.001	0.02	
LOI^(a)^	2.1	2.3	42.5	1.5	
Total	99.9	98.4	97.9	100	

^a^Loss on ignition

^b^Portland cement CEM I/42.5 (Na_2_O_eq_ of 0.79%)

^c^Coarse fly ash (FA-c, 3080 cm^2^/g)

^d^Limestone (Omya, Durcal 5)

^e^Metakaolin (Burgess)

^f^Micro silica (Grade 983-U, Elkem Materials)

Concrete samples were made of different cement pastes and with U aggregates. The effect of aggregate type was investigated for the PC and the LC^3^ samples, using three ASR-reactive aggregates including U, B, P aggregates from Switzerland [30], Bend (Ben) aggregate from the US, and a limestone-based non-ASR reactive aggregate (Cal) (Table 1). The chemical and mineralogical composition of the aggregates based on XRF and X-ray Powder Diffraction (XRD) analyses are shown in Table 3.

**Table 3:** Chemical and mineralogical composition of the ASR-reactive aggregates ((U, B, P, data from [30]) and Bend aggregates) and the calcite-based non-ASR reactive (Cal) aggregates using XRF and XRD (wt%)

Technique	Component	U	B	P	Bend	Cal	
XRF (wt%)	SiO_2_	64.3	69.1	68.0	–		
	Al_2_O_3_	8.8	14.3	7.2			
	CaO	8.7	2.9	8.9			
	K_2_O	2.1	3.4	2.2			
	MgO	2.1	1	1.9			
	Fe_2_O_3_	2.0	2.3	1.4			
	Na_2_O	1.7	3.7	1.4			
	SO_3_	0.4	0.1	0.1			
	LOI^(a)^	9.5	2.6	8.7			
XRD (wt%)	Quartz: SiO_2_ [ICSD 174]	49.7	24.9	55.5	8.6		0.8
	Cristobalite: SiO_2_ [ICSD 75300]	–			1.9		–
	Feldspar: Albite: NaAlSi_3_O_8_ [ICSD 87657]	17.7	33.3	8.2			
	Feldspar: Albite: NaAlSi_3_O_8_ [ICSD 37653]	–			3.7		
	Feldspar: Microcline: KAlSi_3_O_8_ [ICSD 83531]	7	11.7	7.9			
	Feldspar: Orthoclase: KAlSi_3_O_8_ [ICSD 9543]	–	5.9	–			
	Feldspar: Anorthoclase (Na,K)AlSi_3_O_8_ [ICSD 9000857]	–			41.7		
	Mica: MuscoviteKAl_2_(AlSi_3_O_10_)(OH)_2_ [ICSD 75952]	8.5	10.7	7.4			
	Smectite: Vermiculite (Mg,Fe,Al)_3_((Al,Si)_4_O_10_)(OH)_2_.4H_2_O	–			5.8		
	Calcite: CaCO_3_ [ICSD 73446]	6.8	10.4	15.2	–		91.2
	Dolomite: CaMg(CO_3_)_2_ [ICSD 66333]	6.5	0.3	4.5	–		
	Chlorite: Clinochlore Mg_5_Al(AlSi_3_O_10_)(OH)_8_ [ICSD 66258]	0.8	2.4	–			
	Amorphous/non-crystalline	3	0.4	1.3	38.3		8

^a^Loss on ignition

Eight size fractions of aggregates were sieved for U, B, P, and Cal aggregates (Table 4), washed with tap water, and dried at 80 °C for 2 days as described in [30]. The size fraction of concrete samples made of Bend aggregate was 0–4 mm. 1770 kg/m^3^ of aggregate was used to cast the concrete and mortar samples with the cement content of 410 kg/m^3^. Aggregates and cement were mixed for 2 min, tap water was added during half-minute mixing, and the procedure was followed by 3 min of mixing. Concrete samples were cast with a water to binder ratio of 0.46. For the samples with Li, the required amount of LiCl was dissolved in the mix water to obtain 400 mmol/L of Li before mixing. The same procedure was done for the samples boosted to 1.09% Na_2_O_eq_ by adding NaOH.

**Table 4:** Size fractions of U, B, P and Cal^(a)^ aggregate

Size fraction (mm)	Wt%
0.16–0.32	5
0.32–0.63	5
0.63–1.25	5
1.25–2.50	10
2.5–5.0	15
5.0–8.0	15
8.0–12.5	20
12.5–22.4	25

^a^Calcite-based non-ASR reactive

### Mathods

#### Pore solution measurements

Paste samples were cast with a water to binder ratio of 0.46 or 0.6; the higher w/b samples were prepared to allow the collection of pore solution from paste samples after several months. The paste and concrete samples were cast in polypropylene containers with the volume of 200 ml (approximate diameter 55 mm and height 80 mm) after mixing and stored at different temperatures (20 and 40 °C). The pore solutions of the paste and concrete samples were extracted using a compression-testing machine at room temperature. The force used was variable for different paste samples from 300 to 900 kN, and from 1400 to 2200 kN for concrete samples.

The extracted pore solution was immediately filtered after extraction using a 0.2 µm nylon microfilter. In order to measure pH, almost 2 ml of the solution was put in a small plastic container and voltage and temperature were measured after 2 min of equilibration time with a pH electrode (BlueLine 14 pH, SI Analytics)) connected to a Lab 850 pH meter and at the laboratory temperature of ≈ 25 °C. The mV readings were calibrated against potassium hydroxide solutions as detailed in [30, 31]. The pH values are reported at laboratory temperature (25 °C). At high Na or Li concentrations, too low pH readings result due to the so-called alkali error [31], which was corrected based on reference measurements carried out with KOH, NaOH, and LiOH solutions: The pH value was corrected by + 0.2 for 200 mmol/L and by + 0.3 for 300 mol/L of Na and by + 0.35 for 200 mmol/L Li and by + 0.7 for 400 mol/L of Li, comparable to the differences reported in [31].

The remaining solution was immediately diluted 3 or 10 times using ultra-pure water, and stored in the fridge until measuring using Inductively Coupled Plasma Optical Emission Spectrometry (ICP–OES (Shimadzu ICPE–9000)), or Ion Chromatography (IC, Thermo Scientific Intergrion HPIC or Dionex DP ICS-3000 ion chromatograph).

#### Expansion measurements

The samples for the expansion tests with a dimension of 7 × 7 × 28 cm^3^ (three prisms for each mix) were cast in metal molds (lubricated with oil). At both ends of each mold, stainless pins were inserted for measuring the length of the prisms. The molds were covered with a plastic sheet to avoid evaporation, and were demolded after 24 h of casting. The simplified pore solution for each mix was prepared based on the measured pore solution of the corresponding concrete samples after 1 or 2 months, following the procedure outlined in [8, 29]. The following elements were considered to prepare 5 L of the simplified pore solution: Al, K, Na, Ca, and sulfate. The simplified pore solutions were made of de-ionized water and aluminum chloride anhydrous powder (99%, Aesar), calcium sulfate dehydrate (Roth /Merck), sodium sulfate (99%, ACROS), calcium chloride (≥ 98%, RDTH), potassium hydroxide (Sigma Aldrich, 90%) and sodium hydroxide (ACS and ≥ 98%, RDTH). For the system containing Li, LiCl (99.9%, Apollo Scientific) was added to the water to reach 400 mmol/L of Li, and for the boosted system, the required amount of NaOH was added to the water to reach 1.09% Na_2_O_eq_.

For some concretes, the composition of the simplified poresolution was estimated: the simplified poresolution of concrete PC 95% + MK 5% corresponded to measured poresolution of concrete PC 100%, the simplified poresolution of concrete samples PC 90% + MK 10% and PC 80% + MK 20% was prepared based on the measured poresolution of PC 85% + MK 15% with U aggregate.

The expansion was measured on three concrete prisms for each mix, which were immersed, after 24 h of casting, in a box containing 5 L of their own simplified pore solution and were stored at different temperatures (40 and 60 °C). Each box was sealed with plastic foil on top of the box before closing its lid to avoid evaporation. The solution level was kept constant by adding extra water after the monthly measurement.

The length and the weight of each prism were recorded (after drying with a piece of tissue paper) as a function of time. The percentage of length change was calculated based on the following equation:

1 $$ \frac{\Delta L}{L}\left( \% \right) = \frac{Lt - L0}{{L0}} \times 100 $$

where *Lt* is the measured length at desired time and *L*0 is the initial length before expansion (after 3 h of immersion in the simulated solution). For each mix, the mean value was calculated considering the percentage length change of all 3 specimens and the error bars calculated based on the standard deviation of the recorded values.

## Results

### The effect of paste composition on the pore solution composition of paste samples (*W*/*B* = 0.6)

**Fig. 1:**
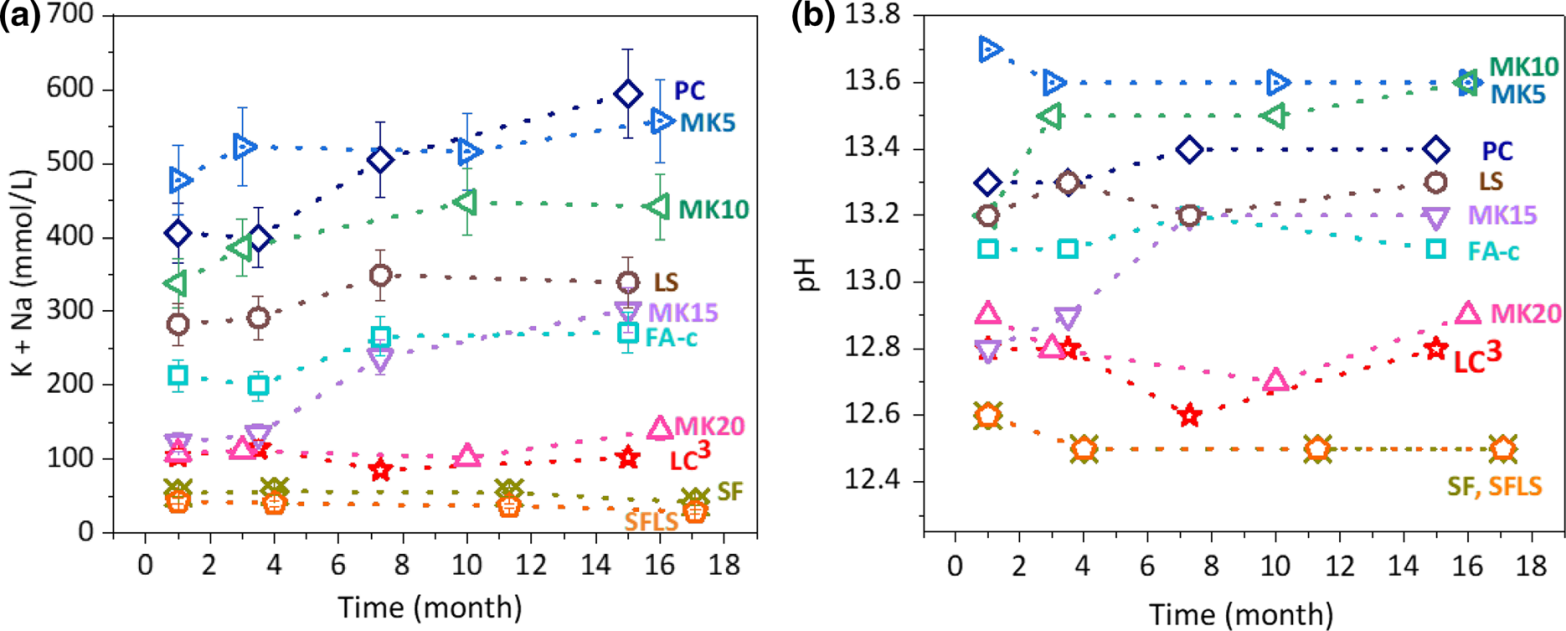
**a** The amount of alkalis (mmol/L) in the extracted pore solution from different pastes (*W*/*B* = 0.6) (the error bars indicate the 10% measurement error of IC/ICP-OES) and **b** the pH values as a function of time at 40 °C

The solution compositions were studied for paste samples stored at 20 °C and 40 °C. Figure 1 shows (a) the amount of alkalis (mmol/L) in the pore solution expressed from different pastes (*W*/*B* = 0.6) and (b) the measured pH values as a function of time at 40 °C. As one set of the expansion tests were done at 40 °C, the results of the poresolution extraction are shown at the same temperature. All measured concentrations are detailed in Table 5.

**Table 5:** Elemental concentrations (mmol/L) and pH values in the pore solution from different paste samples at different temperatures (°C)

Paste (wt%)	W/B	Storage temperature (°C)	Time (month)	K (mmol/L)	Na (mmol/L)	Al (mmol/L)	Ca (mmol/L)	Sulfate ^(a)^ (mmol/L)	Si (mmol/L)	pH ^(b)^	Pressure (KN)	Technique
PC 100	0.6	20	1	316	89	0.68	1.37	2.46	0.35	13.3	300	ICP-OES
			3.5	302	86	0.02	1.83	2.92	0.02	13.4	400	
			7.3	408	114	0.17	1.70	8.43	0.15	13.4	800	IC
			15	405	124	0.07	1.73	8.79	< LOQ ^(c)^	13.4	900	
		40	1	315	91	0.16	1.90	14.90	0.14	13.3	400	ICP-OES
			3.5	305	95	0.02	1.74	17.85	0.02	13.3	400	
			7.3	394	111	0.12	1.74	27.04	0.10	13.4	800	IC
			15	457	137	0.02	3.29	28.68	< LOQ	13.4	900	
	0.46		1	527	129	0.32	1.58	35.63	0.27	13.4	400	IC
			3	528	134	0.10	1.55	39.70	0.13	13.4	450	
PC 95 + MK 5	0.6	20	1	261	136	0.04	2.28	0.97	< LOQ	13.3	800	IC
			3	310	182	0.06	2.50	2.56	< LOQ	13.6		
			10	343	199	0.07	1.92	7.06	0.15	13.6		
			16	345	196	0.08	1.86	7.99	< LOQ	13.6		
		40	1	310	167	0.01	3.96	13.36	< LOQ	13.7	800	IC
			3	328	195	0.05	2.58	19.64	< LOQ	13.6		
			10	325	191	0.04	2.03	20.08	0.04	13.6		
			16	352	206	0.09	2.12	35.94	< LOQ	13.6		
PC 90 + MK 10	0.6	20	1	177	100	0.77	1.12	0.47	< LOQ	13.2	800	IC
			3	191	121	0.76	1.41	0.76	< LOQ	13.2		
			10	211	133	0.40	2.00	1.34	0.20	13.2		
			16	208	140	0.25	1.45	1.87	< LOQ	13.2		
		40	1	210	128	0.11	1.63	3.45	< LOQ	13.2	800	IC
			3	235	152	0.07	4.64	8.96	< LOQ	13.5		
			10	275	172	0.08	3.14	16.66	0.06	13.5		
			16	267	174	0.18	2.63	19.92	< LOQ	13.6		
PC 85 + MK 15	0.6	20	1	128	45	1.72	0.73	0.52	0.35	13.0	700	ICP-OES
			3.5	135	55	0.68	0.70	0.45	0.02	13.1		
			7.3	166	66	1.50	0.90	0.87	0.25	13.1	800	IC
			15	177	75	0.46	1.00	0.72	< LOQ^(c)^	13.3	900	
		40	1	92	32	2.80	0.59	9.00	0.58	12.8	700	ICP-OES
			3.5	97	38	1.59	0.71	9.99	0.54	12.9	700	
			7.3	170	67	0.56	0.95	4.37	0.22	13.2	400	IC
			15	214	89	0.12	2.41	5.80	< LOQ	13.2	400	
PC 80 + MK 20	0.6	20	1	90	62	1.90	1.15	0.35	0.08	13.0	800	IC
			3	86	63	2.45	1.27	0.36	0.08	12.9		
			10	90	68	1.51	0.92	0.58	0.22	13.0		
			16	93	79	1.42	1.05	0.78	0.17	13.0		
		40	1	62	46	2.44	0.99	1.46	0.18	12.9	800	IC
			3	64	48	3.34	0.89	7.92	0.29	12.8		
			10	55	47	2.54	0.60	6.60	0.38	12.7		
			16	77	63	1.70	1.17	3.65	0.24	12.9		
PC 70 + FA 30	0.6	20	1	234	72	0.16	2.99	0.97	0.07	13.2	400	ICP-OES
			3.5	172	62	0.62	0.97	0.95	0.02	13.2	400	
			7.3	213	73	0.48	1.31	2.08	0.25	13.2	800	IC
			15	219	78	0.83	1.68	1.38	< LOQ	13.2	900	
		40	1	161	52	0.67	1.07	2.46	0.29	13.1	400	ICP-OES
			3.5	144	55	0.02	0.97	13.52	0.02	13.1	400	
			7.3	196	70	0.89	0.80	6.04	0.30	13.2	800	IC
			15	196	75	0.51	1.11	3.72	< LOQ	13.1	900	
LC^3^ PC70MK15LS15	0.6	20	1	77	34	1.82	0.76	0.37	0.25	12.9	500	ICP-OES
			3.5	81	35	0.90	0.89	0.29	0.02	12.9	600	
			7.3	106	45	1.95	0.91	0.76	0.27	12.9	800	IC
			15	108	47	1.98	0.98	0.57	0.04	13	900	
	0.6	40	1	72	33	2.99	0.59	4.81	0.37	12.8	500	ICP-OES
			3.5	80	36	1.28	0.66	3.81	0.02	12.8	600	
			7.3	58	27	3.08	0.40	12.41	0.49	12.6	400	IC
			15	70	33	2.42	0.54	5.98	0.21	12.8		
	0.46		1	59	23	3.3	0.44	12.12	0.44	12.5	400	IC
			3	59	24	2.6	0.66	13.37	0.51	12.5	450	
PC 70 + LS 30	0.6	20	1	215	65	0.02	1.51	1.03	0.02	13.3	300	ICP-OES
			3.5	208	66	0.02	2.94	1.55	0.02	13.3	400	
			7.3	286	84	0.08	2.53	3.90	0.09	13.3	800	IC
			15	287	92	0.03	4.16	2.85	< LOQ^(c)^	13.3		
		40	1	218	65	0.02	2.32	5.40	0.02	13.2	300	ICP-OES
			3.5	220	71		3.13	7.55	0.02	13.3	400	
			7.3	267	82	0.25	1.89	7.60	0.14	13.2		IC
			15	257	82	< LOQ	3.39	5.62	< LOQ	13.3		
PC70LS15SF15	0.6	20	1	27	25	< LOQ	14.83	0.24	0.04	12.7	500	IC
			4	16	18		5.92	0.10	0.06	12.5		
			11.3	11	14		8.27	0.13	0.08	12.4		
			17.1	9	12		9.61	0.21	< LOQ	12.5		
		40	1	20	23	< LOQ	7.26	0.34	0.12	12.6	500	IC
			4	17	24		3.46	0.39	0.21	12.5		
			11.3	14	23		7.23	0.42	0.10	12.5		
			17.1	11	19		8.21	0.36	< LOQ	12.5		
PC 85 + SF 15	0.6	20	1	40	34	< LOQ^(3)^	7.76	0.24	0.09	12.7	500	IC
			4	26	27		0.18	0.26	8.64	12.5		
			11.3	23			0.20	0.22	7.46	12.4		
			17.1					0.30	13.21	12.4		
		40	1	26	29	< LOQ	6.37	0.36	0.11	12.6	500	IC
			4	25	32		0.24	0.63	4.64	12.5		
			11.3	23			0.41	0.65	3.95	12.5		
			17.1	18	25		2.43	0.48	0.33	12.5		

^a^Sulfate concentrations were measured using IC for all samples

^b^The pH values were measured and reported at 25 °C

^c^LOQ = Limit of Quantification. LOQ (Al, Na and Si) = 0.02 mmol/L; and LOQ (Ca, K and Sulfate) = 0.01 mmol/L

At 40 °C (see Table 5 for results at 20 and 40 °C), the PC showed alkali concentrations up to 600 mmol/L and correspondingly high pH values of 13.4. A moderate increase of pH from 13.2 at early times to 13.4 after 6 months and longer was observed, which is related to a decrease in the amount of poresolution and due to the continued reaction of clinker which releases alkali to the pore solution [32]. Dilution of the PC with 30 wt% of limestone lowered the alkali concentrations by 200 mmol/L and the pH by 0.2 pH units. The blending with 30 wt% of FA lowered the pH by 0.3 pH units, and with 30 wt% of MK + LS (LC^3^) by 0.6 pH units indicating some reaction of the FA and MK. Increasing amounts of MK lowered successively the pH to 12.9 at 20% MK. The lowest amount of alkalis and lowest pH of 12.5 were observed for the silica fume containing samples SF and SFLS. The high efficiency of the silica-rich SCMs to lower the pH is related to the formation of additional C–S–H, which can bind alkalis lowering alkali concentration in the pore solution. Lower Ca/Si C–S–H can bind more alkalis lowering the pH [33, 34], which explains the lower pH values in the presence of fast-reacting SF and MK compared to slowly reacting fly ash.

These measured results are in agreement with previous reports, which showed that the alkali concentration in the poresolution depends on the amount of alkali in SCMs and on the amounts of SCM used [4, 35–37].

**Fig. 2:**
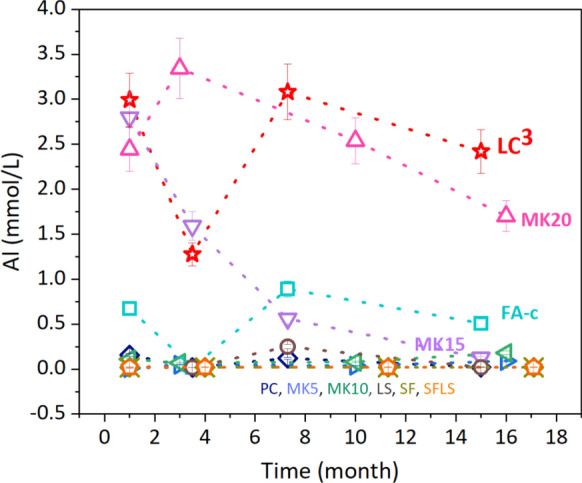
The concentration of Al (mmol/L) in the pore solution of different pastes (*W*/*B* = 0.6) as a function of time at 40 °C. The error bars indicate the 10% measurement error of IC/ICP-OES

Figure 2 shows that in most samples the Al concentrations were around 0.1 mmol/L or below. However, the presence of fly ash and metakaolin increased the Al concentrations; the LC^3^ blend with 15% MK had the highest Al concentration with ≈ 3 mmol/L in the pore solution. This is in agreement with the literature, where an increase in Al concentration in the pore solution was measured in blended cement pastes with Al-rich SCMs [8, 9]. A comparison of the measured Al concentrations at 40 °C with those at 20 °C (see Table 5) shows that an increase in temperature increases the amount of Al in the pore solution. Based on the observation in PC and FA blended cements [10, 38, 39] a further increase of Al concentration can be expected for samples exposed at 60 °C.

### Expansion of concrete samples

#### Effect of cement

**Fig. 3:**
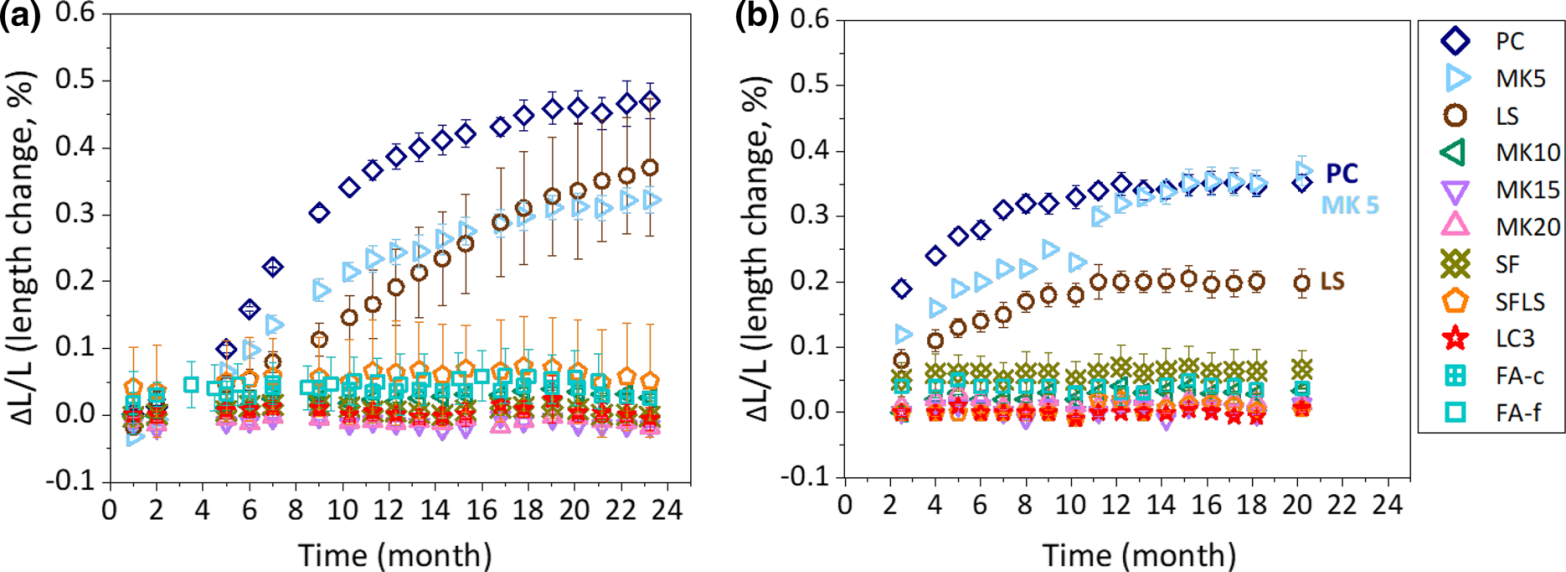
The extent of ASR expansion of concrete prisms made of different pastes and U aggregate (*W*/*B* = 0.46) as a function of time **a** at 40 °C and **b** at 60 °C. The error bars are the standard deviation of the recorded values

Figure 3 shows the extent of ASR expansion of concrete prisms made of different pastes using the U aggregate (*W*/*B* = 0.46) as a function of time (a) at 40 °C and (b) at 60 °C.

Concrete prisms made of PC showed the fastest and highest expansion at 40 and at 60 °C. The sample at 60 °C expands faster than that at 40 °C as also reported in the literature [40], although the total expansion after long times is higher at 40 °C. The lower expansion of the limestone-containing sample (PC 70% LS 30%) can be attributed to the dilution effect. The concrete sample made of PC 95% and MK 5% showed only a slightly slower expansion than the one with plain PC, while the presence of 10, 15, or 20% MK was sufficient to suppress expansion. None of the samples containing FA, MK ≥ 10% and SF showed any expansion up to almost 20–23 months at both temperatures.

#### Effect of extra alkali hydroxide

**Fig. 4:**
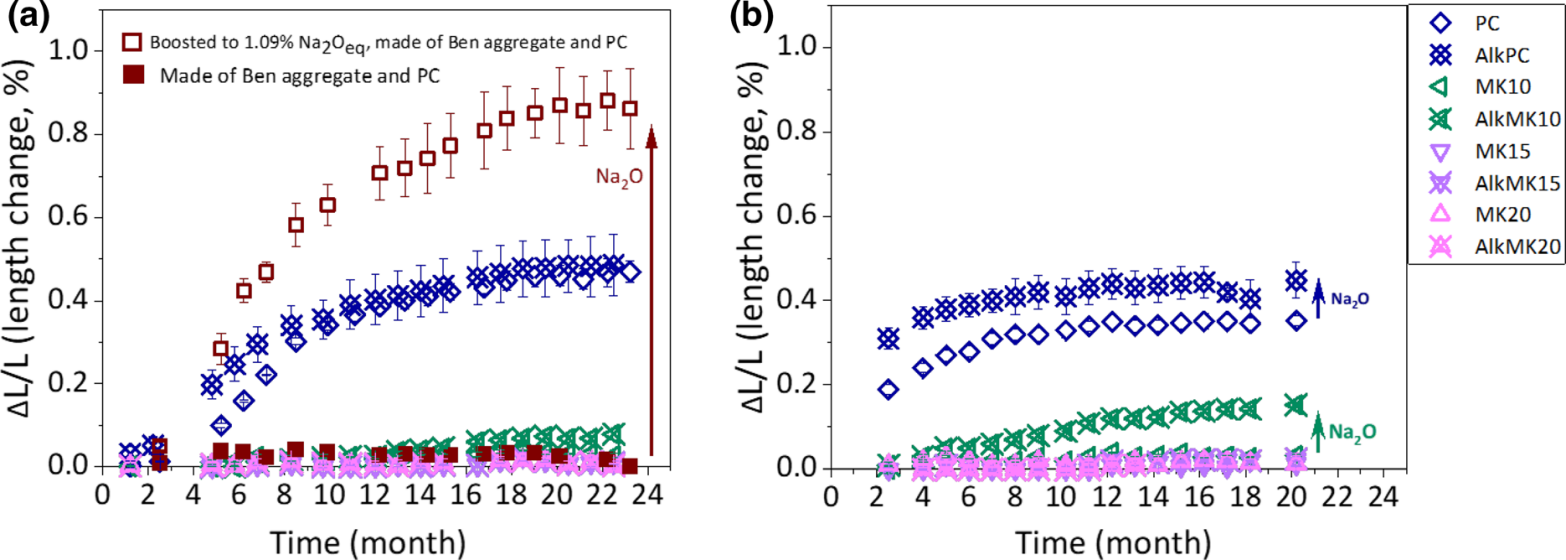
The effect of extra alkali (boosted to 1.09% Na_2_O_eq_ by adding NaOH) on the extent of ASR-expansion for concrete prisms made of different paste and U or Bend aggregate (filled and empty rectangle dots) (*W*/*B* = 0.46) as a function of time **a** at 40 °C and **b** at 60 °C. The error bars are the standard deviation of the recorded values

Figure 4 shows the effect of extra alkali hydroxide (boosted to 1.09% Na_2_O_eq_ by adding NaOH) on the extent of ASR expansion for concrete or mortar prisms.

The addition of extra alkali is expected to increase the dissolution rate of aggregates as well as the extent of ASR formation. In fact, the addition of NaOH (boosted to 1.09% Na_2_O_eq_) to PC slightly increased the extent of expansion at 60 °C, and the rate at 40 °C for the concrete samples made of U aggregate (Fig. 4). The U aggregate concrete samples with 10% MK plus Na_2_O showed some expansion at 60 °C, but not at 40 °C within the time investigated, while samples 15 and 20% MK additions did not expand even with the addition of extra alkali. The samples made of Bend aggregate, which contains 38% of ill-crystalline material (Table 3), showed no expansion for PC (with 0.79% Na_2_O_eq_) but showed a very strong expansion if the boosted to 1.09% Na_2_O_eq_ (by adding NaOH).

#### Effect of lithium

**Fig. 5:**
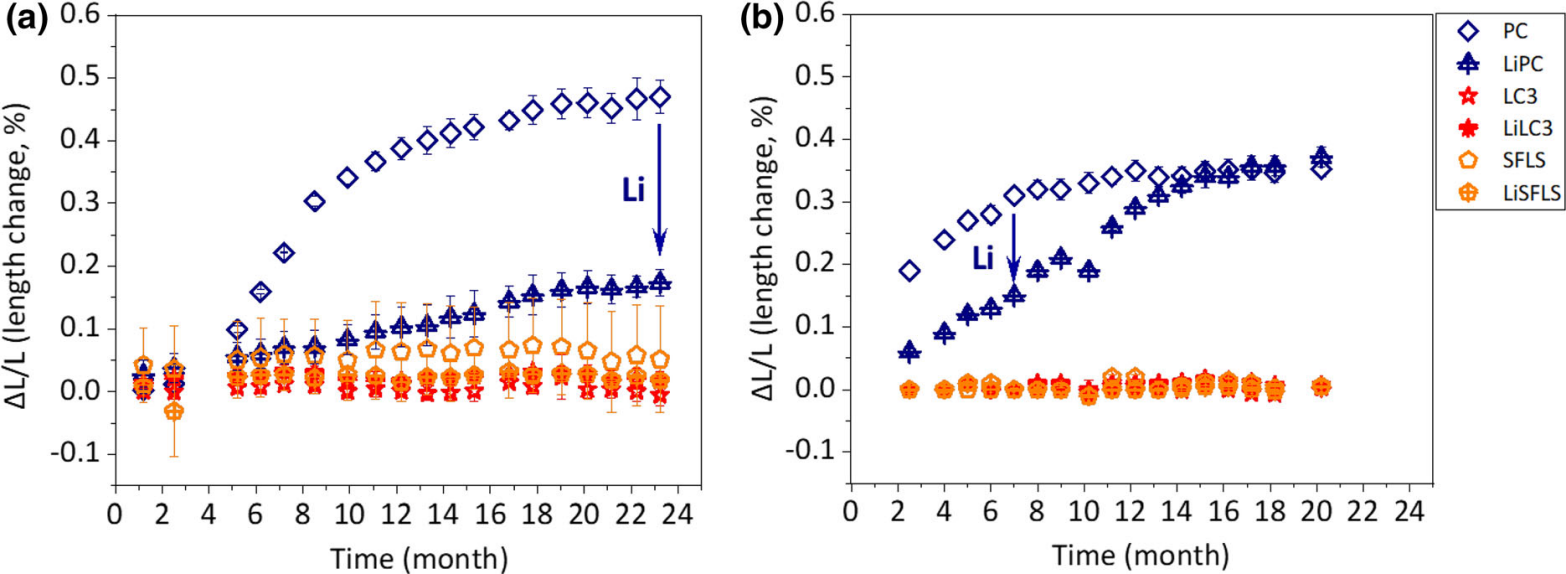
The effect of Li addition (plus 400 mmol/L LiCl) on the extent of ASR-expansion for concrete prisms made of different pastes and U aggregate (*W*/*B* = 0.46) as a function of time **a** at 40 °C and **b** at 60 °C. The error bars are the standard deviation of the recorded values

Figure 5 demonstrates that LiCl addition can slow down and lower the extent of ASR-expansion at 40 °C and at 60 °C.

The addition of 400 mmol/L lithium slowed down the expansion of PC concrete prisms at 40 °C and at 60 °C. However, at 60 °C a similar expansion was observed after 1 year, which could possibly be related to the binding of Li in the formed ASR products, lowering the concentrations of Li in the pore solution with time. In fact, after 1 month only roughly 50% (222 mmol/L Li) of the initially 400 mmol/L Li added was still present in the pore solution (see Table 6, data of sample LiPC).

**Table 6:** pH values and elemental concentrations (mmol/L) in the pore solution from concretes (W/B = 0.46) with U aggregate based on different cements at 40 °C, measured using IC

Paste (wt.%)	Time (month)	K (mmol/L)	Na (mmol/L)	Al (mmol/L)	Ca (mmol/L)	Sulfate (mmol/L)	Si (mmol/L)	Li (mmol/L)	pH ^(a)^	Pressure (KN)
PC 100	1	421	126	0.70	0.86	27.76	0.78		13.3	1800
	3	378	124	0.14	0.55	30.83	3.00		13.2	2000
AlkaliPC100	1	363	318	0.36	1.26	48.12	0.86		13.6	
LiPC	1	393	133	0.13	1.50	29.81	0.41	222	13.3	
PC95 + MK5	1.7	443	159	0.07	2.51	2.00	< LOQ^(b)^		13.7	
PC90MK10	1.7	204	86	0.45	0.86	3.57	0.18		13.2	
AlkaliPC90MK10	1	154	240	3.60	0.37	17.48	1.10	0.68	13.4	
PC85MK15	1	86	70	1.83	0.44	2.37	0.52		12.9	
	4	84	71	1.58	0.60	3.28	0.42		12.9	
PC80MK20	1.7	75	42	2.29	0.58	2.50	0.40		12.8	
AlkaliPC80MK20	1	59	131	6.93	0.24	26.30	1.06		12.7	
LC^3^ (PC70MK15LS15)	1	76	36	2.1	0.58	3.10	0.40		12.8	
	3	70	37	1.2	0.63	5.30	0.49		12.7	
PC70FA30	1	172	112	0.50	0.70	6.17	0.69		13.1	
	4	144	103	0.43	0.79	7.63	0.83		13.0	
PC70LS30	1	230	161	0.05	1.63	9.46	0.13		13.5	
	4	187	155	0.09	0.98	12.29	0.97		13.4	
PC70SF15LS15	1	32	32	0.04	4.46	11.71	0.08		12.4	
	4	40	38	0.07	2.33	15.72	0.11		12.5	
PC85SF15	1	57	49	0.05	2.18	13.37	0.19		12.6	
	4	66	59	0.1	2.08	15.69	0.13		12.7	

^a^The pH values were measured at 25 °C

^b^LOQ = Limit of Quantification. LOQ (Al, Na and Si) = 0.02 mmol/L; and LOQ (Ca, K and sulfate) = 0.01 mmol/L

### The effect of aggregate mineralogy on solution composition of PC and blended cements

**Fig. 6:**
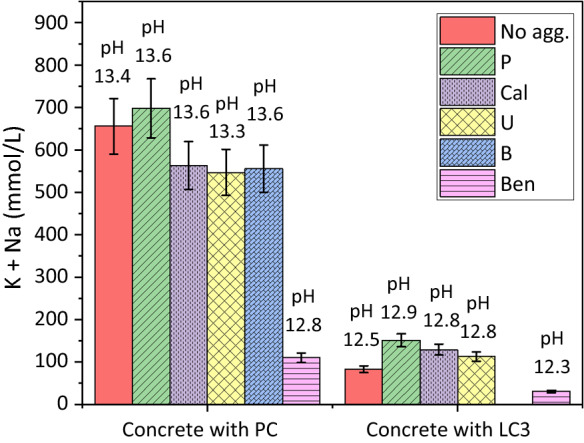
The amount of released alkalis (mmol/L) from PC or LC^3^ pastes (No agg.) and different concretes made of PC or LC^3^ and different aggregates (*W*/*B* = 0.46); and pH values after 28 days at 40 °C. The error bars indicate the 10% measurement error of IC

Figure 6 shows the total Na + K concentrations (in mmol/L) and pH values in the pore solution of PC or LC^3^ pastes and of concretes containing different aggregates. The presence of aggregates might increase the alkali concentration in the poresolution due to the release of alkali present in aggregate. In contrast, the binding of alkali by C-S–H [33, 34] or on the surface of silica or due to the formation of ASR products, which contain significant amounts of alkali [41, 42], are expected to lower the alkali concentration in the pore solution.

The pore solutions of the PC concrete made of P aggregate had a comparable alkali concentration as the PC paste, while Cal, U, and B had slightly lower alkali concentrations in comparison with the PC paste although within the error of measurement (Fig. 6). The presence of additional NaOH (boosting to 1.09% Na_2_O_eq_) in concrete made of U aggregate increased to total alkali concentration by ≈ 140 mmol/L (+ 0.3 pH units, see Table 6, sample labeled AlkaliPC100), leading to a faster and slightly higher expansion (see Fig. 4).

The PC sample with Bend aggregate showed a 500 mmol/L lower alkali concentration and a decrease of pH by 0.8 pH units compared to the paste sample indicating a strong binding of alkalis, most probably by additional C–S–H containing alkalis [43, 44]. This strong decrease of pH and alkali concentrations in the pore solution indicates a strong reaction of the Bend aggregate, in contrast to the Cal, U, P, and B aggregate. The observed strong decrease in pH and alkali concentrations could explain the so-called pessimum effect (= low ASR expansion) observed for fast-reacting [43, 44] and very fine aggregates [45, 46]. In fact, the samples made of PC and Bend aggregate showed no significant expansion (Fig. 4a). The presence of additional NaOH (boosting to 1.09% Na_2_O_eq_) increased the total alkali concentration by ≈ 115 mmol/L (+ 0.4 pH units, see Table 7, data of AlkaliPC and Ben aggregate).

**Table 7:** pH values and elemental concentrations (mmol/L) in the pore solution from concretes (W/B = 0.46) at 40 °C prepared with different aggregates, measured using IC

Paste (wt.%)	Aggregate	Time (month)	K (mmol/L)	Na (mmol/L)	Al (mmol/L)	Ca (mmol/L)	Sulfate (mmol/L)	Si (mmol/L)	pH ^(a)^	Pressure (KN)
PC 100	U	1	421	126	0.70	0.86	27.76	0.78	13.3	1800
		3	378	124	0.14	0.55	30.83	3.00	13.2	2000
	P	1	496	202	0.17	0.93	51.83	1.36	13.6	
		3	474	206	0.36	0.17	53.80	12.46	13.6	
	B	1	346	210	0.55	0.87	22.71	1.10	13.6	
		4	375	241	0.25	0.75	38.03	0.96	13.6	
	Cal	1	347	217	0.10	1.21	21.80	0.04	13.6	
		4	379	246	0.19	1.15	34.77	< LOQ^(b)^	13.6	
	Ben	1	17	93	0.03	3.82	0.53	0.03	12.8	
		4	22	108	0.08	1.93	1.31	0.10	12.8	2200
AlkaliPC		1	48	175	0.19	0.57	2.55	1.27	13.2	2000
LC^3^ PC70MK15LS15	U	1	76	36	2.10	0.58	3.10	0.40	12.8	1400
		3	70	37	1.22	0.63	5.30	0.49	12.7	2000
	P	1	109	42	2.32	0.32	4.99	1.09	12.9	
		3	114	43	1.99	0.56	5.96	0.74	12.9	
	Ben	1	8	22	0.85	4.12	0.66	0.24	12.3	
		3	7	20	0.75	2.40	0.63	0.20	12.2	2200
	Cal	1	87	42	2.01	0.29	2.98	0.64	12.8	1800
		3	93	46	1.85	0.49	3.44	0.41	12.9	2000

^a^The pH values were measured at 25 °C

^b^LOQ = Limit of Quantification. LOQ (Al, Na and Si) = 0.02 mmol/L; and LOQ (Ca, K and Sulfate) = 0.01 mmol/L

For LC^3^ based samples, lower pH values in the range of 12.3–12.9 were observed. The pore solutions of concrete samples made of P, Cal, and U aggregates had slightly higher alkali concentrations compared to the paste, and again lower alkali concentrations were measured for the concrete made of Bend aggregate, indicating additional alkali binding in the case of Bend aggregate.

**Fig. 7:**
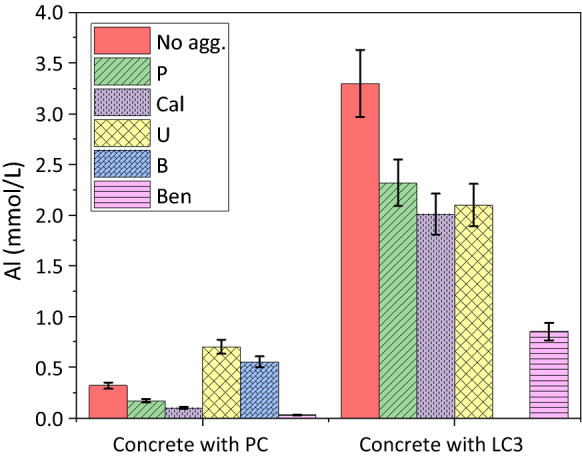
The Al concentration (mmol/L) of the extracted pore solution from PC or LC^3^ pastes (No agg.) and different concrete samples made of PC or LC^3^ and different aggregates (*W*/*B* = 0.46) after 28 days at 40 °C. The error bars indicate the 10% measurement error of IC

Figure 7 and Tables 6 and 7 show that the aggregates also affect the Al concentration in the extracted pore solution from PC or LC^3^. The concentration of Al in the pore solution is relatively low for PC paste and all concrete samples made of PC. The maximum Al concentration was observed for concrete samples made of U and B aggregates, while the Al-concentration in the Bend sample was low. All three aggregates, U, B and Bend, have substantial amount of feldspars, such that no clear effect of the feldspar content on the Al content in poresolution was observed.

The lower Al concentration could be related to the lower pH value in the presence of Ben aggregate, as the Al-concentration in the pore solution of concrete and cements increases with pH [4, 47].

For the paste and concretes made of LC^3^ higher Al concentrations are observed. The presence of aggregates somewhat lowered the Al concentrations for the Bend aggregate. For older samples (3–4 months after casting), the same trends of alkali and Al concentrations were measured for all samples made of PC or LC^3^ pastes and concretes (e.g. Table 6). It was shown that in the presence of 1 mmol/L of Al the dissolution rate of silica (quartz & amorphous silica) at 40 °C decreased by a factor of 10 at pH 13 (pH referring to 20 °C), and by a factor of 2 at pH 13.5 [28]. Thus, in particular, for concrete samples made of LC^3^, the Al concentration in the pore solution can be expected to slow down the dissolution of the aggregates and thus ASR formation in the long term.

The addition of lithium had no significant effect on pH values nor on the concentration of the other elements in the pore solution (Table 6, sample labeled LiPC).

### Effect of pore solution on expansion

**Fig. 8:**
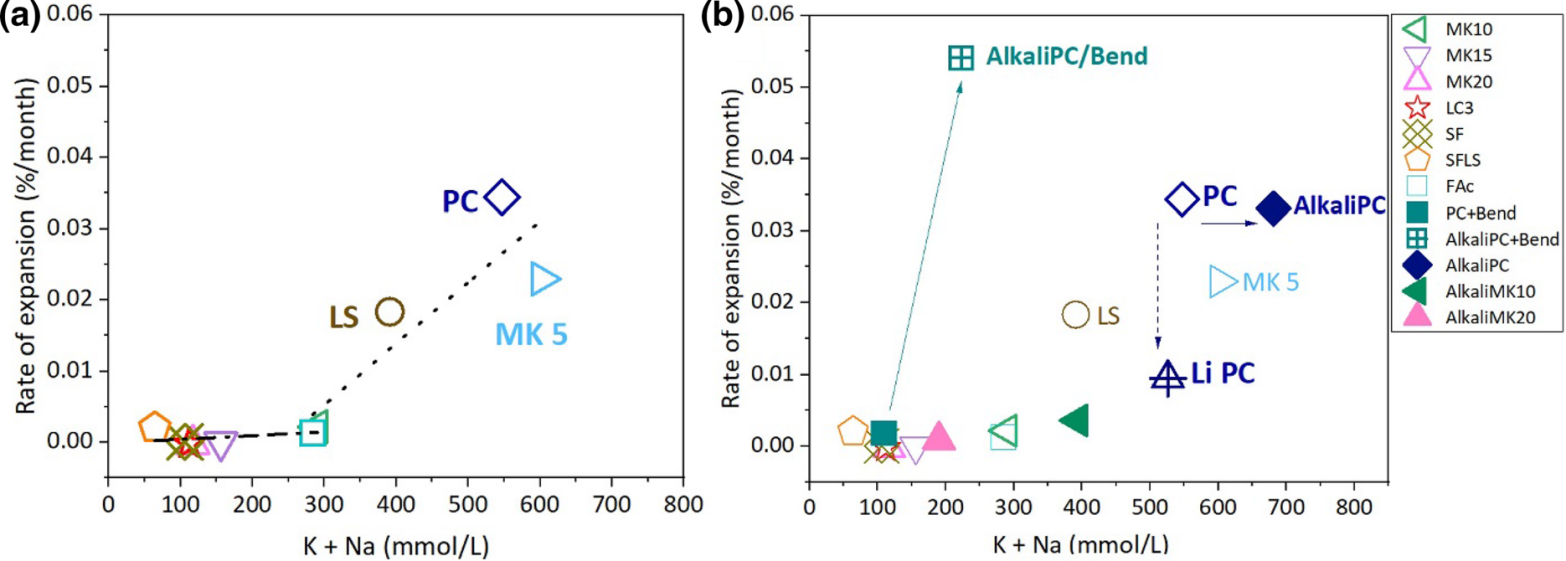
The rate of expansion (%/month) as a function of K + Na (mmol/L) in the extracted pore solution from different concretes made of **a** U aggregate and **b** U or Bend aggregate (*W*/*B* = 0.46) after 1/1.7 months and at 40 °C. Arrows indicate the effect of alkali boosting, dashed arrows the effect of Li

The rates of expansion for concrete samples (%/month) were calculated from the slope of the expansion curves shown in Fig. 8 as a function of time at 40 °C up to 15 months. Based on the observation that the pore solution composition did not change noticeably after the first month, the alkali content of the pore solution was taken after 1 month (for MK5, 10, and 20 after 1.7 months; Tables 6 and 7). As the expansion was slightly slower after 15 months probably due to some leaching, and also the pore solution results were considered at early age, the expansion rates were calculated with the results up to 15 months.

Figure 8a shows that the samples did not significantly expand during 15 months in poresolutions with less than 300 mmol/L K + Na and pH ≤ 13.2. Only above this threshold, significant expansion was observed and the rate of expansion increased exponentially with pH and alkali concentration.

For concrete samples made of U aggregate, as the cement blended with metakaolin shows increased Al concentration, in particular, MK 15 and MK20 (see Table 6), it was expected that this would lower ASR expansion. However, due to the low alkali content in the pore solution little expansion was observed for SF and MK samples during the 15 months of expansion measurement. Dedicated dissolution experiments on silica and feldspar at high pH values revealed that Ca and sulfate can increase dissolution rate [28]. In fact, the more expanded samples, PC, MK 5, and LS, showed higher Ca and sulfate concentrations than the other samples (with exception of containing SF concretes; Table 6). However, no clear correlation was observed between the expansion of concrete made of U aggregate and Al, Ca or sulfate concentrations. Thus, the expansion of concretes made of U aggregate can be explained purely based on the pH values and the total alkali concentration, Fig. 8a.

The presence of additional NaOH (boosting to 1.09% Na_2_O_eq_) increased the total alkali concentration by ≈ 140 mmol/L or 0.3 pH units (see Table 6) for the concretes made of U aggregate, but increased the measured expansion only moderately for the PC and PC90MK10 and not significantly for the samples with more MK (> 10%) and thus lower pH values, Fig. 8b.

For the sample with Bend aggregate (Fig. 8b), the boosting to 1.09% Na_2_O_eq_ increased the total alkali concentration by ≈ 115 mmol/L (Table 7, data of AlkaliPC and Ben aggregate) similar to the PC concrete with U aggregate (Table 6, sample labeled PC100). Boosting, however, resulted in a strongly increased expansion for concrete containing the Bend aggregate, which could be to the presence of very reactive aggregates in this sample.

The addition of LiCl slowed down the rate of expansion (Fig. 8b), although no changes in the total alkali concentration or pH was measured (Table 6, sample labeled LiPC), indicating that this lower expansion rate is probably due to changes in ASR product.

## Conclusions

Measurement of ASR expansion of concrete samples using different aggregates and different cements, showed the highest expansion rate for concretes made of Portland cement only. A faster expansion was observed at 60 °C than at 40 °C. Dilution of PC by blending with 30% of limestone decreased the expansion by 30%. Blending with fly ash, metakaolin (≥ 10%) and micro-silica were more efficient in suppressing expansion due to ASR, and none of these samples showed expansion neither at 40 nor at 60 °C up to almost 2 years.

Boosting the samples with NaOH accelerated the expansion clearly at 60 °C, while little effect was observed at 40 °C. The addition of 400 mmol/L LiCl slowed down the expansion rate at 40 °C and at 60 °C, although at 60 °C after 1 year and longer a similar expansion was observed in the absence and presence of Li, which might be related to the uptake of Li in the hydrates formed with time.

The composition of the pore solution composition was affected strongly by the composition of the SCM used. Dilution of the PC with 30 wt% of limestone lowered the pH by 0.2 pH units, blending with 30 wt% of FA by 0.3 pH units, and with MK + LS (LC^3^) by 0.6 pH units. The lowest pH was observed for the silica fume-containing samples indicating a higher efficiency of the silica-rich SCMs to lower the pH. Also, the aggregate composition affected the alkali content of the pore solution. The very reactive Bend aggregate, which contains mainly feldspar, lowered the alkali concentrations in the pore solution by 80% after 1 and 4 months, indicating a very significant uptake of K and Na in the reaction products formed.

A clear trend between Na + K concentration in the pore solution and ASR expansion was observed; the highest extent of expansion was measured for the concrete samples with the highest alkali and hydroxide concentrations. No clear correlation between expansion and aluminium, calcium, or sulfate concentration was observed. Thus, based on the present study, high temperature and pH are the main drivers of ASR reaction.

## Figures and Tables

**Fig. 9:**
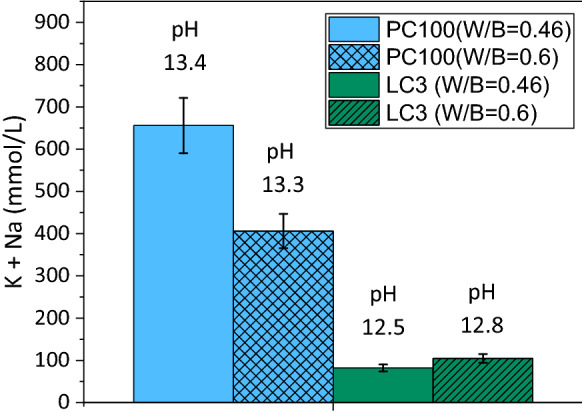
The amount of released alkalis (mmol/L) from PC and LC^3^, with *W*/*B* = 0.46 and 0.6 after 28 days and at 40 °C

## Appendix A

### The influence of water to binder ratio on the alkali content in the pore solution

Figure 9 illustrates that lower water to binder ratio, as used in the concrete experiments, increases the amount of alkalis (mmol/L) in the pore solution from 400 to 650 mmol/L in the case of PC, due to the lower amount of solution available and due to the high Ca/Si C–S–H formed in PC, which limits alkali binding by C–S–H [33, 34]. In contrast, little effect on the alkali concentrations was observed for the LC^3^ cement paste, where more C–S–H is present with a lower Ca/Si of ≈ 1.6 [48], which together buffers the alkali concentration to a constant value of ≈100 mmol/L. The same trends of alkali concentration and pH values on the pore solution were observed after 3.5 months for PC and 3 months for LC^3^ (Table 5).
